# Suburbanisation of oral cavity cancers: evidence from a geographically-explicit observational study of incidence trends in British Columbia, Canada, 1981–2010

**DOI:** 10.1186/s12889-015-2111-9

**Published:** 2015-08-08

**Authors:** Blake Byron Walker, Nadine Schuurman, Ajit Auluck, Scott A. Lear, Miriam Rosin

**Affiliations:** Department of Geography, Simon Fraser University, 8888 University Drive, Burnaby, BC V5A 1S6 Canada; British Columbia Cancer Agency, Vancouver, BC Canada; Department of Biomedical Physiology and Kinesiology, Simon Fraser University, Burnaby, BC Canada; Faculty of Health Sciences, Simon Fraser University, Burnaby, BC Canada; Division of Cardiology, Providence Health Care, Vancouver, BC Canada; Department of Pathology and Laboratory Medicine, University of British Columbia, Vancouver, BC Canada

## Abstract

**Background:**

Recent studies have demonstrated an elevated risk of oral cavity cancers (OCC) among socioeconomically deprived populations, whose increasing presence in suburban neighbourhoods poses unique challenges for equitable health service delivery. The majority of studies to date have utilised aspatial methods to identify OCC. In this study, we use high-resolution geographical analyses to identify spatio-temporal trends in OCC incidence, emphasising the value of geospatial methods for public health research.

**Methods:**

Using province-wide population incidence data from the British Columbia Cancer Registry (1981–2009, *N* = 5473), we classify OCC cases by census-derived neighbourhood types to differentiate between urban, suburban, and rural residents at the time of diagnosis. We map geographical concentrations by decade and contrast trends in age-adjusted incidence rates, comparing the results to an index of socioeconomic deprivation.

**Results:**

Suburban cases were found to comprise a growing proportion of OCC incidence. In effect, OCC concentrations have dispersed from dense urban cores to suburban neighbourhoods in recent decades. Significantly higher age-adjusted oral cancer incidence rates are observed in suburban neighbourhoods from 2006 to 2009, accompanied by rising socioeconomic deprivation in those areas. New suburban concentrations of incidence were found in neighbourhoods with a high proportion of persons aged 65+ and/or born in India, China, or Taiwan.

**Conclusions:**

While the aging of suburban populations provides some explanation of these trends, we highlight the role of the suburbanisation of socioeconomically deprived and Asia-born populations, known to have higher rates of risk behaviours such as tobacco, alcohol, and betel/areca consumption. Specifically, betel/areca consumption among Asia-born populations is suspected to be a primary driver of the observed geographical shift in incidence from urban cores to suburban neighbourhoods. We suggest that such geographically-informed findings are complementary to potential and existing *place-specific* cancer control policy and targeting prevention efforts for high-risk sub-populations, and call for the supplementation of epidemiological studies with high-resolution mapping and geospatial analysis.

## Background

Globally, oral cavity cancers (OCC) are the 10^th^ most common cancers among males and 18^th^ among females, accounting for an estimated 299 051 new cases in 2012 [1]. Significant inequalities in OCC incidence have been observed [2, 3], reflecting variations in known risk factors, specifically age, ethnicity, and tobacco, alcohol, and betel/areca nut consumption [4–8]. Recent studies have confirmed significantly higher incidence [3, 6, 9, 10], prevalence [10], mortality [11], and lower survival [12, 13] among socioeconomically deprived populations. Socioeconomic deprivation can be defined as a state of disadvantage resulting from a combination of social, economic, and situational influences on an individual, neighbourhood, or community. While the literature on socioeconomic deprivation and OCC incidence continues to mature, no studies to date have contrasted patterns of cancer incidence between urban, suburban, and rural neighbourhoods [14]. This geographical differentiation may reveal unique risk profiles useful for informing cancer control policy. Accordingly, this study utilises geospatial methods to analyse and map spatio-temporal trends in OCC, characterising findings using unique local geographies of suburbanisation, deprivation, and demography. In this way, we provide a template for geospatially-informed epidemiological analysis of cancer registry data.

In the post-World War II period in North America, a move to the suburbs signified a rise in social class as people left the deprived inner-cities for more affluent neighbourhoods [15, 16]. However, the last two decades have witnessed the suburbanisation of socioeconomically disadvantaged populations in North America and Western Europe [14, 17–20]. The health risks of suburban life are well documented, with researchers demonstrating links between adverse health outcomes, reduced access to health care resources relative to urban cores [18], more sedentary lifestyles [21], and lower community cohesion [22] among suburban residents. Residential population density has also been linked to poor health [23, 24], with a substantial literature from the 1990s exploring the hypothesis that dense urban areas somehow contributed to higher cancer incidence [25, 26] and mortality [27], although these studies are typically conducted for large areal units (e.g., at the city scale, rather than the neighbourhood), and none to date have focussed on OCC.

The objective of this analysis was to identify geographical trends in OCC incidence in British Columbia from 1981 to 2009, with a focus on suburban growth and socioeconomic deprivation. Accordingly, we sought to (1) map concentrations of OCC cases over space and time, (2) compute and contrast age-adjusted incidence rates between urban, suburban, and rural residents over time, and (3) characterise these trends by the local socioeconomic, demographic, and cultural characteristics of areas with high a concentration of cases.

## Methods

### Ethics statement

Ethics approval for this study was obtained from the University of British Columbia/British Columbia Cancer Agency Research Ethics Board (H08-00839) and the Simon Fraser University Research Ethics Board (2013s0753).

### Data

Census population data (years 1981, 1986, 1991, 1996, 2001, and 2006) within British Columbia for the smallest-area geographical units (approximately 85 residents per unit) were obtained from Statistics Canada. Male and female populations for each geographical unit and each census year were mapped using geographical information systems and the population density for each census geographical unit was calculated. The proportion of residents aged 65 years and over was also calculated for every census geographical unit. Results were manually cross-checked against Statistics Canada records for verification. Each geographical unit was then classified as urban, suburban, or rural, based on its population density, using Statistics Canada’s definition of suburban neighbourhood as a Census area with an average population density between 150 and 400 persons per square kilometre [28]. This metric was selected for consistency with official Statistics Canada and Organisation of Economic Co-operation and Development data and literature.

Oral cavity cancer incidence data were acquired from the British Columbia Cancer Registry, a comprehensive population-based provincial registry. Data included all cases from 1981 to 2009 (inclusive) with International Classification of Diseases in Oncology (version 3) site codes C003-5 (mucosa of upper and lower lips), C020-23 (dorsal surface, ventral surface, border and anterior 2/3^rd^ of tongue), C028-29 (overlapping lesions of tongue and tongue), C030-31, 039 (upper and lower gum), C040, 041, 048,049 (anterior, lateral floor of mouth, overlapping lesions of floor of mouth, floor of mouth), C050-52,058, 059 (soft palate, hard palate and uvula, overlapping lesions of palate, palate), and C060-62, 068,069 (cheek, vestibule of mouth, retromolar area, mouth, and unspecified parts of the mouth) [5]. Data fields comprised patient age at the time of diagnosis, patient sex, patient residential postal code at the time of diagnosis, and year of diagnosis (aggregated to 5-year periods corresponding to the aforementioned census years).

### Spatial and statistical analyses

Using geographical information systems, each case was mapped by patient residential postal code at the time of diagnosis. To visualise the geographical distribution of incidence, case locations were spatially interpolated using the kernel density estimation method [29]. This method constructs a spatial density function around each point on the map and produces a visual hotspot, such that areas with many cases are brighter than areas with few or no cases.

Each case was placed on the neighbourhood type map of its respective census year, corresponding to the year of diagnosis. In this way, we derived the neighbourhood type (urban, suburban, or rural) of each case at the time of diagnosis.

Pearson’s Chi-square test for association was used to determine whether neighbourhood type (rural, suburban, or urban) is associated with 5-year period and patient sex. Trends in the proportion of cases in each neighbourhood type were evaluated using the Cochrane-Armitage Chi-square test. The mean case population density was calculated for each neighbourhood type in each 5-year period. To examine the geographical relationships between incidence, age, and ethnicity, the percentage of population aged 65 and over and the percentage of population born in India, China, or Taiwan (regions with high betel quid/areca nut consumption) were mapped for each census geographical unit.

Age-adjusted incidence rates (AAIRs) with 95 % CI were calculated for each neighbourhood type and 5-year period, using the 1996 British Columbia standard population (selected because it was the midpoint of the study period). Patients under age 40 years were excluded (*n* = 41, 0.7 %) to minimise estimate error induced by low case counts in younger populations. Due to data incompleteness for the year 2010 we projected case counts for that year, assuming an equal distribution of the annual number of cases from 2005 to 2009 (i.e., the average AAIR per year from 2005 to 2009 was added to the four-year rate to simulate the year 2010).

To investigate the temporal trends in neighbourhood types, 5-year periods, sex, and socioeconomic deprivation, we used the Vancouver Area Neighbourhood Deprivation Index (VANDIX). The VANDIX score was calculated for each patient postal code using data from the 2006 census, as described in our previous work [30, 31]. Median VANDIX scores for each neighbourhood type were calculated to examine trends in patient neighbourhood deprivation.

## Results

OCC cases were mapped and classified by 5-year period and neighbourhood type (*N* = 5473). The resulting case counts are shown in Table 1. A greater overall proportion of male cases (64 %) is observed throughout, a finding consistent with the literature [11]. However, this disparity is decreasing as female patients comprise a growing proportion of OCC incidence. The proportion of female cases in suburban areas has doubled since the first 5-year period (from 2.3 % of total incidence in 1981–85 to 5.3 % in 2005–09); conversely, the proportion of male suburban cases appears to be in decline (from 6.6 % of total incidence in 1981–85 to 5.5 % in 2005–09).

**Table 1:** Number of Cases by Year, Sex, and Neighbourhood type

	1981-85		1986-90		1991-95		1996-00		2001-05		2006-09		
	Male	Female	Male	Female	Male	Female	Male	Female	Male	Female	Male	Female	Total
Urban	355	113	482	242	508	312	459	328	466	300	317	261	4143
Suburban	42	15	55	22	63	35	43	29	53	35	44	43	479
Rural	85	28	107	39	121	47	83	40	97	62	97	45	851
Total by sex	482	156	644	303	692	394	585	397	616	397	458	349	5473
Total by period	638		947		1086		982		1013		807		

**Fig. 1:**
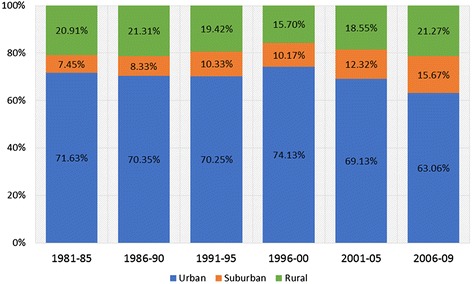
Proportion of cases by neighbourhood type. Urban cases decline and rural cases remain relatively stable while the proportion of suburban cases doubles from 1981 to 2009. These trends are statistically significant for all three neighbourhood types (*p* < 0.01)

Significant increases in the proportion of suburban cases (Cochrane-Armitage *x*^2^ = 418.144, df = 1, *p* < 0.0005) and rural cases (Cochrane-Armitage *x*^2^ = 9.458, df = 1, *p* < 0.002) were detected, as shown in Fig. 1; the proportion of urban cases declined over the years, which was also found to be highly significant (Cochrane-Armitage *x*^2^ = 123.064, df = 1, *p* < 0.0005).

**Fig. 2:**
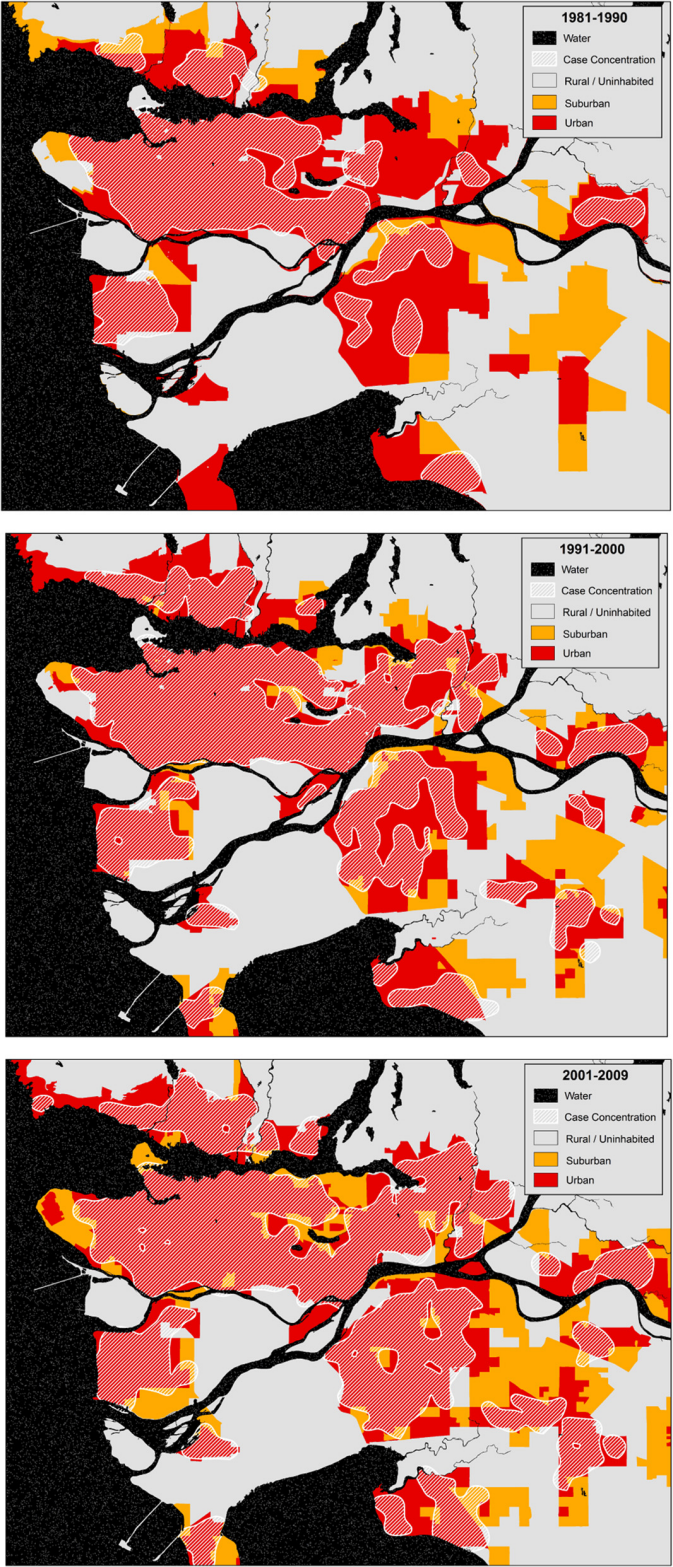
Oral cavity cancer case concentrations for Vancouver, by decade. Case concentrations (approximated by white hashed areas) are found to disperse from the urban cores in the 1980s to the surrounding areas in the 1990s and 2000s, including into lower-density suburban areas. This pattern is consistent throughout cities in British Columbia

When mapped, temporal trends in case concentrations are visible from 1981 to 2009, as shown for the Metro Vancouver area in Fig. 2. White pixels represent a concentration of cases within 1500 m. In the first decade 1981–1990, cases were concentrated in urban areas, dispersing throughout the 1990s and 2000s into the suburban fringe. While only Metro Vancouver is shown in this figure, similar patterns are found in all other cities in British Columbia. These maps are not published to protect patient confidentiality.

**Fig. 3:**
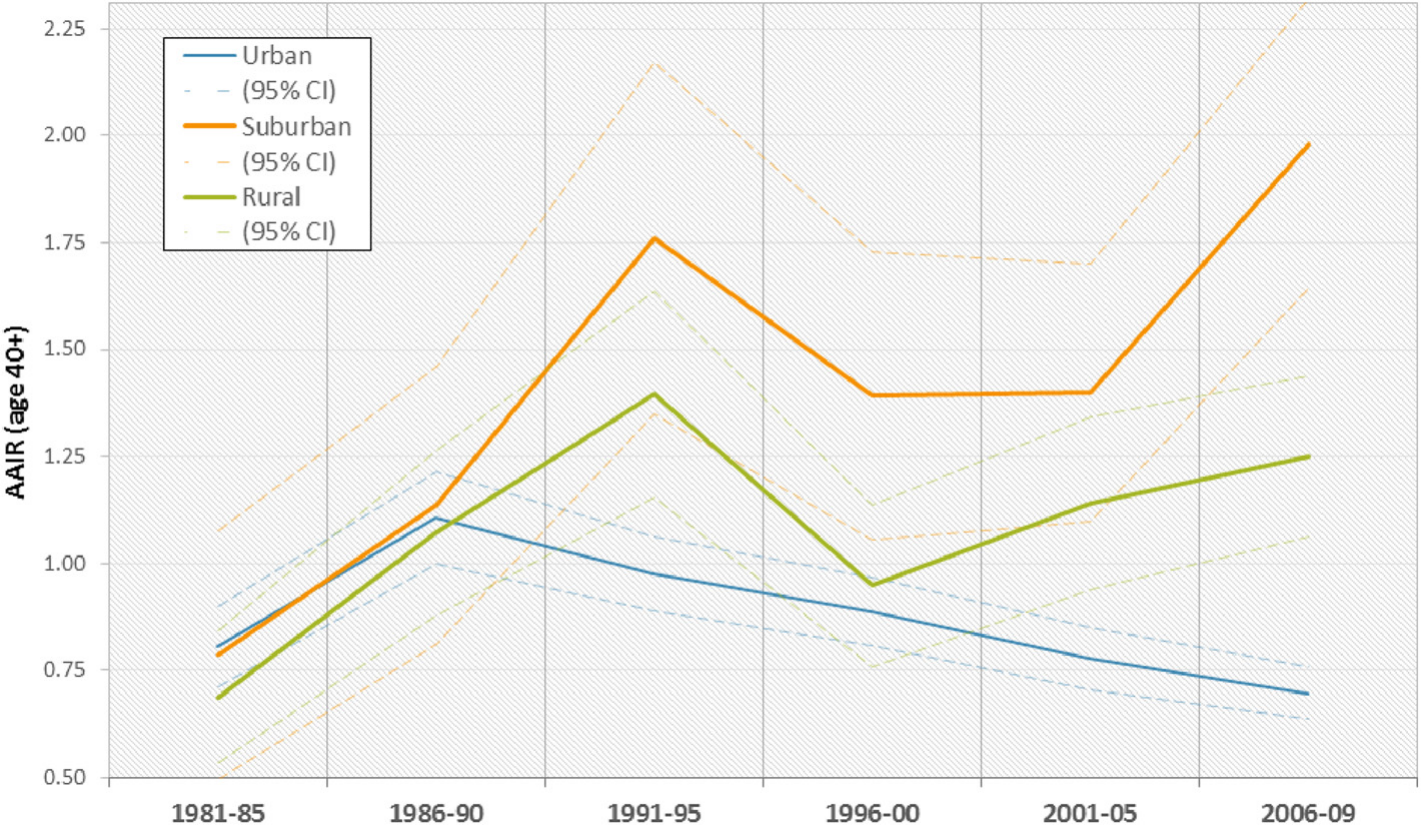
Age-adjusted incidence rates (per 500 000 person-years, in five-year intervals) by neighbourhood type, illustrating the shift in incidence from urban cores outwards to the suburbs

AAIRs for OCC are shown in Table 2 and Fig. 3. A divergence between urban, suburban, and rural incidence rates is observed following the 1986–1990 5-year period. The steady decline in urban rates is contrasted by increases in rural and suburban incidence. The divergent trend in age-adjusted incidence rates confirms the observed trend of fewer case concentrations in dense urban cores accompanied by increasing incidence in rural/suburban areas, supporting our mapped findings.

**Table 2:** Age-Adjusted Incidence Rates by Neighbourhood Type, per 500 000 Person-Years, with 95 % Confidence Intervals

	1981-85	1986-90	1991-95	1996-00	2001-05	2006-09
Urban	0.81 (0.71, 0.90)	1.11 (1.00, 1.21)	0.98 (0.89, 1.06)	0.89 (0.81, 0.97)	0.78 (0.70, 0.85)	0.70 (0.64, 0.76)
Suburban	0.79 (0.49, 1.08)	1.14 (0.81, 1.46)	1.76 (1.35, 2.17)	1.39 (1.06, 1.73)	1.40 (1.10, 1.70)	1.98 (1.64, 2.32)
Rural	0.69 (0.54, 0.84)	1.07 (0.88, 1.26)	1.40 (1.16, 1.64)	0.95 (0.76, 1.14)	1.14 (0.94, 1.34)	1.25 (1.06, 1.44)

**Fig. 4:**
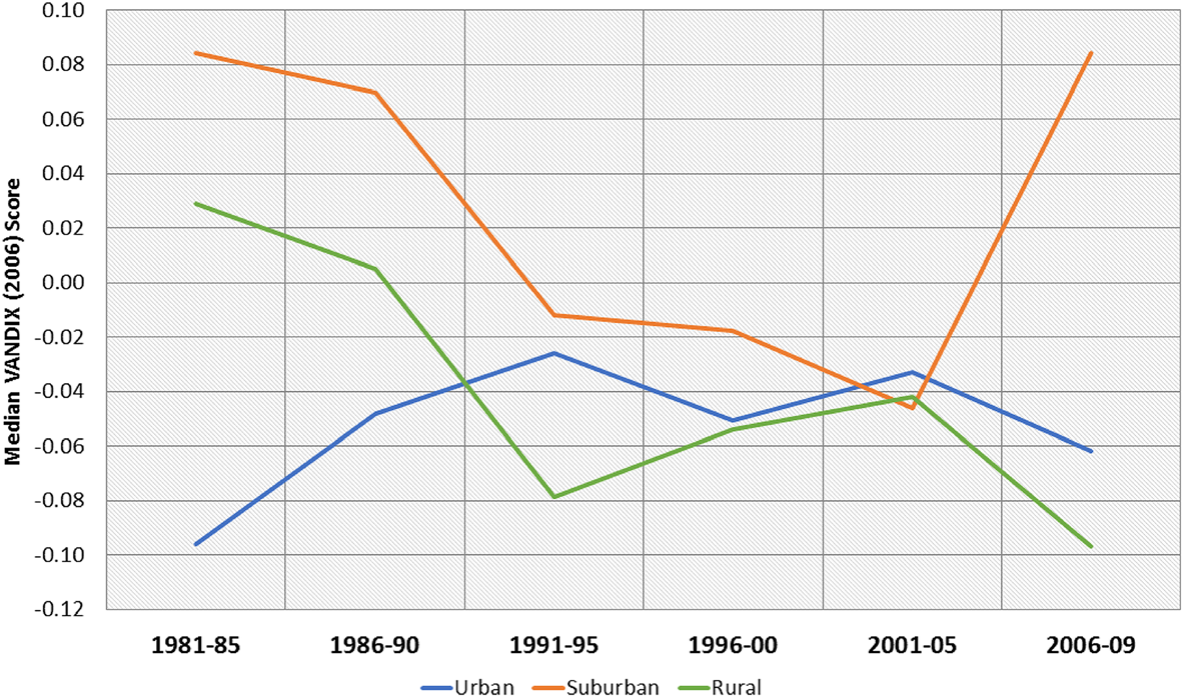
Median VANDIX score by neighbourhood type. Note the convergence in recent decades, interrupted by a sharp rise in deprivation among suburban patients from 2006 to 2009. This may reflect the documented increase in suburban deprivation and its known correlation with OCC incidence

The Vancouver Area Neighbourhood Deprivation Index scores for each patient’s residential postal code at the time of diagnosis show distinct trends between neighbourhood types, as shown in Fig. 4. Higher levels of socioeconomic deprivation within suburban and rural patients’ census areas were observed in the 1980s. However, the 2005–09 period is characterised by a sharp increase in suburban patients’ median neighbourhood deprivation score, corresponding to declining SES among OCC patients.

**Fig. 5:**
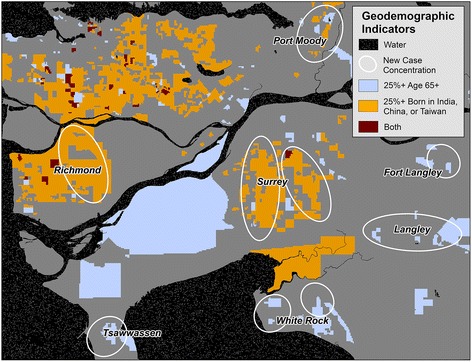
Metro Vancouver 2006 census geographical units where over 25 % of residents are ages 65+ and/or born in India, China, or Taiwan. New oral cancer case concentrations since the 1990s are approximated by white ellipses, found exclusively on the urban periphery. The observed increase in age-standardised incidence rates in suburban areas may be explained by the high percentage of immigrant populations from India, China, and Taiwan, where consumption of betel quid/areca nut is high

Neighbourhoods with a high proportion of seniors correspond to case concentrations in most cities in the study area, as shown in Fig. 5. However, case concentrations not coincident with a high proportion of people ages 65+ years appear to have high proportions of Indian, Chinese, or Taiwanese residents; very few neighbourhoods have both.

## Discussion

Through the use of spatio-temporal mapping and geospatial analysis, this study provides novel insight into rising OCC incidence in suburban neighbourhoods. By mapping these data, we identified divergent trends in suburban areas otherwise obscured in epidemiological studies using an urban/rural dichotomy. Through examination of the resulting trends and maps, we hypothesise that this recent increase in both un-adjusted and adjusted incidence in suburban neighbourhoods may be explained by three simultaneous geodemographic transitions in the suburbs including: increases in aging; socioeconomic deprivation; and increases in betel/areca consuming populations.

As established in the literature, risk behaviours such as tobacco and alcohol consumption correlate with socioeconomic deprivation among oral cancer cases [32]; this pattern is particularly strong in our study findings. However, the association between suburban incidence and deprivation may be linked to changes in the age structure and ethnic composition of suburban areas in British Columbia. Previous research has documented increasing deprivation levels among suburban immigrants in Canada, suggesting that foreign-born residents bear a disproportionate burden of socioeconomic disadvantage [19, 20].

In 2011, one in every four British Columbians was foreign-born, 72 % of whom arrived from Asia (primarily India, China, and Taiwan) [33]. Previous studies have found disproportionately high oral cancer rates among South Asian and Chinese populations [34], including a study conducted in British Columbia [5]. While tobacco use is very prevalent (particularly among males) in China, studies have shown significantly lower use among Chinese and South Asian populations in Canada than other ethnic groups [35, 36]. Lower consumption of alcohol among Chinese and South Asian populations in Canada was also observed [35]. However, these ethnic groups have a high prevalence of betel quid and areca nut consumption (with or without tobacco) [36]. A recent meta-analysis of fifty publications implicates betel quid and areca nut in half of all oral cancer incidence in India (49.5 %, chewed with tobacco) and Taiwan (53.7 %, chewed without tobacco) [37]. In the Southern provinces of China, the prevalence of betel/areca use is as high as 82.7 %, although data for most regions are highly limited [38]. While traditionally rare in North America, there is a high prevalence of betel/areca consumption among South Asian, Chinese, and Taiwanese immigrants in Western regions, including in British Columbia [34, 39, 40].

The observed development of OCC case concentrations in areas of high East- and South-Asian immigration may be due to several combinations of risk factors, including betel/areca consumption (with and without tobacco), socioeconomic deprivation, and aging among immigrant populations. Similar demographic transitions are occurring also in the United States and Western Europe, driving increased betel/areca-related oral cancer incidence [37]. The use of map-based and spatial-analytical studies in these regions may yield additional evidence to inform this hypothesis and inform place-specific public health interventions.

While the majority of cases occur in urban areas throughout the study period, the proportion of cases in suburban areas has more than doubled since 1981. This emergence of suburban incidence is observed in cities throughout the province and may be due to a growing senior population in these previously rural areas. While in 1991 the average proportion of residents ages 65 years and over in suburban neighbourhoods throughout British Columbia was 13.1 %, it has steadily risen to 15.5 % in 2006. Conversely, the urban average in 1991 was 17.7 %, falling to 14.7 % in 2006 and rural figures have remained around 12 % throughout. This transition suggests an increased overall burden of oral cancer risk in suburban areas and may partially explain the observed geographical pattern shown in Fig. 2.

The recess in age-adjusted incidence rates observed in the 5-year period 1996–2000 may be attributable to the redrawing of official census area boundaries in 1996. However, by 2006–2009, a clear divergence is apparent between neighbourhood types. That this pattern persists after age-adjustment suggests that the growing senior population in suburban neighbourhoods does not entirely account for the observed increase in suburban oral cancer rates.

A distinct convergence of socioeconomic deprivation in the 1990s and early 2000s may be explained by increasing suburban and rural affluence as baby boomers relocated from urban centres to surrounding areas. However, we hypothesise that the subsequent increase in median suburban deprivation in the most recent decade is linked to suburbanisation of deprivation and is a contributing factor to the increase in suburban incidence in recent decades. That high deprivation is geographically coincident with immigrant neighbourhoods underscores their unique barriers in access to screening and treatment. The challenges imposed by language, mobility, and cultural norms are amplified by suburban deprivation, isolation, and increased travel distance to health care resources [18, 41]. Accordingly, we emphasise the need for improved education and awareness of OCC risk factors such as betel quid/areca nut consumption, and underscore the importance of accessible, culturally-sensitive screening programs in North America, Western Europe, and other high-immigration regions undergoing suburbanisation.

### Study strengths and limitations

The use of geospatial methods enabled us to categorise and map finer-resolution geographical patterns that would have been obscured using traditional epidemiological methods. Additionally, this approach enabled a more nuanced classification to include suburban cases beyond the common urban/rural dichotomy. Through the examination of map-based data, supplemented with our local knowledge of the study area, we hypothesised that immigration and aging patterns may be geographical correlates to increasing suburban OCC incidence. The use of a map-based data analysis platform (geographical information systems) facilitated the investigation of these hypotheses both visually and using statistical methods.

Crucially, population data from the BC Cancer Registry include over 90 % of all known cases, enabling us to infer with a high degree of confidence that the observed trends reflect the true patterns at the population level. Census data for every census year in the study enabled temporally accurate neighbourhood classification, while the Vancouver Area Neighbourhood Deprivation Index provided insight into the socioeconomic context of suburban cancer incidence. However, the use of 2006 census data to model deprivation limits its accuracy for earlier time periods. Additionally, our method for constructing AAIRs for 2010 assumes a rate consistent with the preceding four-year period (2005–2009), and low female incidence prevented the calculation of reliable sex-specific AAIR estimates.

While the suburbanisation of OCC case concentrations was observed for all cities in British Columbia, low numbers of Asia-born immigrants outside the Metro Vancouver area limits our ability to address the betel/areca hypothesis in smaller urban areas. Future map-based analyses may yield more insight into this pattern in the context of other large North American and Western European cities.

## Conclusion

This study has identified a shift in oral cavity cancer incidence from urban cores to the suburbs through recent decades in British Columbia, Canada. This spatial shift is coincident with changes in socioeconomic deprivation associated with urban, rural, and suburban neighbourhoods. Crucially, the higher observed incidence in suburban areas may be explained by an increasing number of senior residents, socioeconomically deprived populations, and patterns of immigrant settlement and associated betel/areca consumption among Asia-born populations. Future research is required in other study areas to identify the extent and magnitude of the patterns observed herein. The findings of this study are directly applicable to public health policy implementation including identification of areas where increased culturally-sensitive screening for OCC may be appropriate.

The growing ubiquity of maps in mobile and web-based applications underscores their potential to communicate spatial knowledge. Geospatial methods, such as those used in this study, enable the spatio-temporal analysis and mapping of cancer registry data to provide researchers with cartographic tools for developing epidemiological hypotheses, identifying opportunities for location-specific policy, and targeting high-risk sub-populations. As such, we advocate for greater use of geospatial methods to supplement traditional epidemiological studies and communicate results to policymakers.
